# Acute diverticulitis: beyond the diagnosis: predictive role of CT in assessing risk of recurrence and clinical implications in non-operative management of acute diverticulitis

**DOI:** 10.1007/s11547-024-01841-8

**Published:** 2024-07-22

**Authors:** Stefania Simonetti, Silvia Lanciotti, Dominga Carlomagno, Flaminia De Cristofaro, Gioacchino Galardo, Bruno Cirillo, Fabio Fiore, Giacomo Bonito, Carola Severi, Paolo Ricci, PDTA Group on Uncomplicated Acute Diverticulitis, Domenico Alvaro, Marco Assenza, Enrico Baldini, Carmen Catero, Emanuela Conti, Giuseppe Donato, Giampiero Ferraguti, Enrico Fiori, Deborah Grilli, Anna Santa Guzzo, Franco Iafrate, Antonella Lamazza, Marco Lucarelli, Andrea Mingoli, Nadia Pallotta, Francesco Pugliese, Laura Zinnamosca

**Affiliations:** 1 Department of Radiology, Oncology and Pathology, Sapienza University of Rome, Viale Regina Elena 324, 00161 Rome, Italy; 2 Emergency Radiology Unit, Policlinico Umberto I Hospital, Viale del Policlinico 155, 00161 Rome, Italy; 3 Translation and Precision Medicine Department, Sapienza University of Rome, Viale del Policlinico 155, 00161 Rome, Italy; 4 Emergency Department, Sapienza University of Rome, Viale del Policlinico 155, 00161 Rome, Italy; 5 UOC Gastroenterologia, University Sapienza, Rome, Italy; 6 UOSD Pronto Soccorso Chirurgico, University Sapienza, Rome, Italy; 7 UOSD PS Medico e OBI, AO Policlinico Umberto I, Rome, Italy; 8 UOSD Risk Management e Audit Clinico, University Sapienza, Rome, Italy; 9 UOC Gastroenterologia, University Sapienza, Rome, Italy; 10 UOS Patologia Clinica - Laboratorio Centrale, University Sapienza, Rome, Italy; 11 UOC Chirurgia Oncologica e Laparoscopica, University Sapienza, Rome, Italy; 12 UOC Radiologia, University Sapienza, Rome, Italy; 13 UOSD Endoscopia Digestiva e Operativa, University Sapienza, Rome, Italy; 14 UOS Patologia Clinica - Laboratorio Centrale, University Sapienza, Rome, Italy; 15 UOC Chirurgia d’Urgenza e del Trauma, University Sapienza, Rome, Italy; 16 UOC Centro di Rianimazione, University Sapienza, Rome, Italy

**Keywords:** Uncomplicated diverticular disease, Hinchey classification, Outpatient management, Integrated care pathway

## Abstract

**Purpose:**

The aim of the study is to identify CT findings that are predictive of recurrence of acute uncomplicated colonic diverticulitis, to better risk-stratify these patients for whom guidelines recommend a conservative outpatient treatment and to determine the appropriate management with an improvement of health costs.

**Materials and Methods:**

Over the past year, 33 patients enrolled in an outpatient integrated care pathway (PDTA) for uncomplicated acute diverticulitis with 1-year follow-up period, without recurrence, and 33 patients referred to Emergency Department for a recurrent acute diverticulitis were included. Images of admission CT were reviewed by two radiologists and the imaging features were analyzed and compared with Chi-square and Student *t* tests. Univariate and multivariate Cox regression models were employed to identify parameters that significantly predicted recurrence in 1-year follow-up period and establish cutoff and recurrence-free rates. The maximally selected rank statistics (MSRS) were used to identify the optimal wall thickening cutoff for the prediction of recurrence.

**Results:**

Patients with recurrence showed a greater mean parietal thickness compared to the group without recurrence (16 mm vs. 11.5 mm; HR 1.25, *p* < 0.001) and more evidence of grade 4 of peridiverticular inflammation (40% vs. 12%, *p* = 0.009, HR 3.44). 12-month recurrence-free rates progressively decrease with increasing thickness and inflammation. In multivariate analysis, only parietal thickness maintained its predictive power with an optimal cutpoint > 15 mm that causes a sixfold increased risk of recurrence (HR 6.22; 95% CI, 3.05–12.67; *p* < 0.001). Beyond thickness and peridiverticular inflammation, predictive value of early recurrence within 90 days from the 1st episode resulted also an Hinchey Ib on admission CT.

**Conclusions:**

The maximum wall thickening and the grade of peridiverticular inflammation can be considered as predictive factors of recurrence and may be helpful in selecting patients for a tailored treatment to prevent the risk of recurrence.

## Introduction

Acute diverticulitis (AD) represents one of the most common gastrointestinal diseases leading to hospitalization in western society. Hospital admissions for diverticulitis have increased 7.5% annually from 190 per 100000 in 2008 to 310 per 100000 in 2015 in Europe [1], resulting in significant economic burden on the healthcare system, both in Europe [2–4] and in USA [5, 6].

Furthermore, these numbers are expected to increase, due to the growing prevalence of this disease, especially among younger patients.

Computed tomography (CT) imaging has become a primary diagnostic tool in the diagnosis and staging of patients with acute diverticulitis. The most used classification is based on the Hinchey criteria [7], modified by the recent German guidelines [8]. 75% of patients develop the uncomplicated form of acute diverticulitis [9]. The classification of uncomplicated forms has an important implication in clinical practice because, based on the recent international guidelines [10–12], a conservative outpatient treatment is recommended. Both an international consensus conference [13] and meta-analyses [14, 15] also suggest that antibiotic therapy is not necessary. With an accurate multidisciplinary management protocol, which provides for the short-term follow-up of the patient, a rapid discharge of patients with a diagnosis of acute uncomplicated diverticulitis would therefore be feasible. The implementation of such a therapeutic approach would considerably reduce healthcare costs [16].

In this context, an integrated care pathway (PDTA) for uncomplicated acute diverticulitis has been organized for outpatient program at Policlinico Umberto I Hospital, Roma, Italy. After diagnosis at the Emergency Department, patients are discharged with observational treatment borne by an expert team of gastroenterologists with medical examination at the outpatient gastroenterological clinic within 72 hours, 30 days and 3 months after discharge. The institution of standardized clinical pathway for acute non-complicated colonic diverticulitis has the following objectives: to abbreviate the stay in Emergency Department of patients, optimizing the time needed for diagnosis; to reduce inappropriate hospitalizations; to provide high quality specialist outpatient care for management and therapy with personalized pathways, ensuring both short- and long-term follow-up; and to ensure a multidisciplinary approach in the clinical management of the patient and the availability of dialogue between healthcare professionals and general practitioners. However, to improve the quality of patient care, it would be useful to establish whose patients are at risks of early and late AD recurrences. Patients with a recurrent attack of diverticulitis may be at risk for developing complications, need for emergency surgery and developing more than one recurrent episode.

In a retrospective cohort study [17], the authors suggested that the severity of acute inflammation and the wall thickening of affected colonic segment may be useful predictors for recurrence, although a cutoff value for parietal thickness was not defined as well as the time of recurrence. No predictive factors were identified by another cohort study [18] while more than five diverticula per 10 cm of colon resulted a significant predictor of recurrence on right AD [19, 20]. Discrepancies among studies could be ascribed to the heterogeneous population of AD patients that have been analyzed.

This study is aimed to identify CT imaging findings that could predict the risk of recurrence within 12 months in a cohort of patients with uncomplicated AD, thereby aiding in selecting patients enrolled in our PDTA who may be suitable for more intensive non-operative treatment to prevent recurrence of the disease.

## Materials and methods

### Study population

This retrospective observational study only used information gathered from human participants, without any identifiers linking individuals to the data/samples. All methods and procedures meet institutional and research committee ethical standards in accordance with the 2013 Declaration of Helsinki.

Over the past year, 66 patients referred to the Emergency Department of Policlinico Umberto I Hospital (Rome) for uncomplicated AD were included. Uncomplicated acute diverticulitis was defined as modified Hinchey stages 1a and 1b; therefore, patients have a small pericolic abscess (< 5 cm) or covered perforation, with the absence of ascites or free abdominal air. Thirty-three of these patients were those enrolled in the outpatient uncomplicated AD care program (PDTA) with 1-year follow-up without recurrence (NR-pts) and 33 patients, matched per gender and age, were patients admitted for a recurrent AD (R-pts) within 12 months from the previous 1st access for AD to the Emergency Department. Patients with recurrence were also divided into early recurrence after first episode of acute diverticulitis (< 90 days, 13 patients) and late recurrence (> 90 days, 20 patients). Exclusions criteria were: high-risk subjects (immunocompromised patients, presence of associated comorbidities), need for emergency surgery and development of complications at 1st episode (Hinchey > 2). All admission 1st episode CT scans were revaluated and examined.

### CT scanning protocol

CT examinations were performed using the two CT scanners of the Emergency Radiology Department: Philips Ingenuity 64-slice and Siemens Somatom Sensation 64-slice, with intravenous iodinated contrast injection, after eGFR (Estimated Glomerular Filtration Rate) evaluation, based on Policlinico Umberto I Hospital Guidelines regarding prevention of acute kidney injury from iodinated contrast media. All protocols included basal phase and arterial, portal, and excretory phases, from the lung base to the pelvis, with coronal and sagittal multiplanar reconstructions, and slice thickness of 5 mm and 15 mm.

### CT imaging analysis

**Fig. 1:**
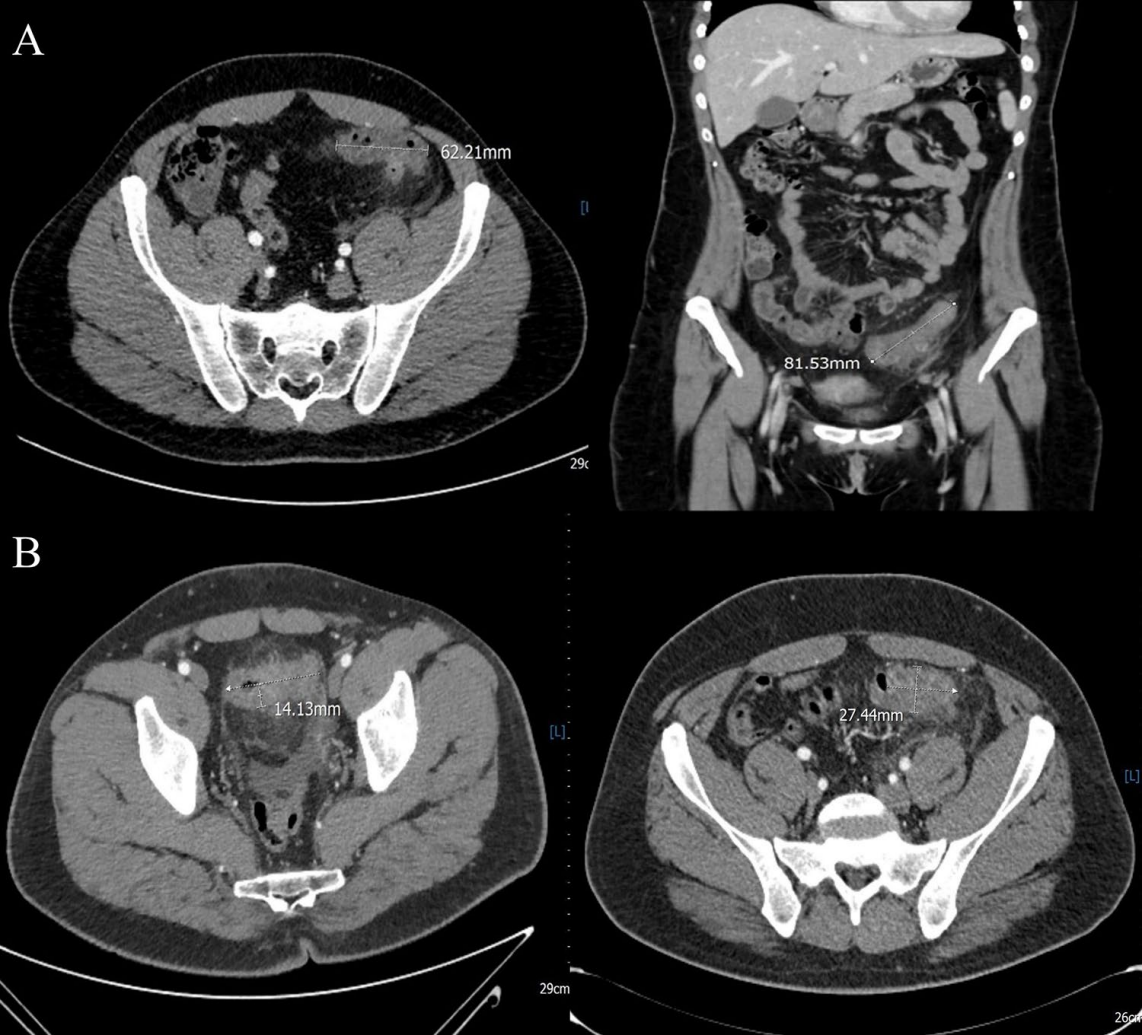
**A** axial and coronal measurement of involved colon segment length; **B** measurement of maximum parietal thickness (serosa-to-mucosal surface) of inflamed segment with visible lumen and measurement of maximum parietal thickness (serosa-to-serosa surface) of inflamed segment without visible lumen

**Fig. 2:**
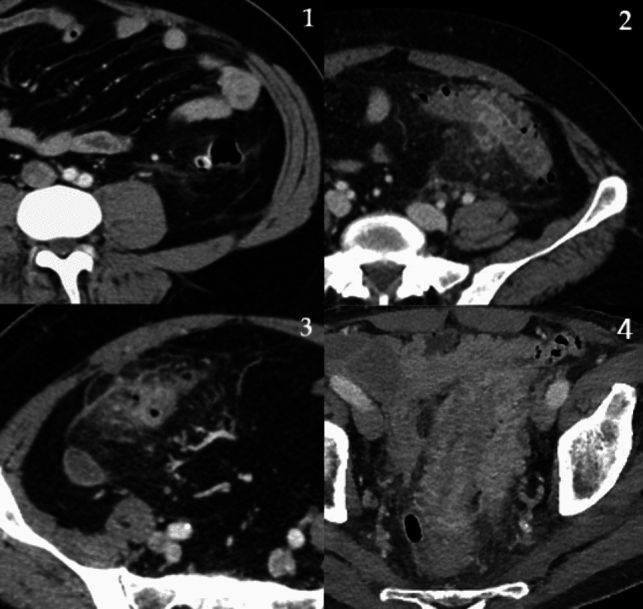
Axial CT showing peridiverticular inflammation grades: (1) Minimal: rare fine threads of high attenuation. (2) Mild: multiple threads of high attenuation that remain distinct, vessels are clearly visible. (3) Moderate: many threads difficult to resolve individually, vessels difficult to discern. (4) Severe: dominant pattern of increased attenuation in the fat could be mistaken for fluid collection, vessels not visible

First episode CT images were analyzed by an emergency radiologist with > 10 years of experience (S.L.) and a fourth-year radiology resident (S.S.). CT scan revaluation and measurements were performed using INFINITT PACS. CT outcome measures were known parameters of disease severity [17–19] : (a) *maximum length of the involved colonic segment:* length of involved colonic segment was measured using both axial and coronal multiplanar reconstructions, based on morphology of segment involved and its rotation on the various planes, using the digital caliber of the PACS system (Fig. 1); (b) *number of diverticula within 10 cm*: first, the colonic segment with the highest concentration of diverticula was identified, and then, the maximum number of diverticula contained within 10 cm of that segment was counted*;* (c) *maximum colonic wall thickness (in millimeters) of the affected segment*: as shown in Dickerson et al. [17], for the correct measurement it is necessary to identify the colonic lumen and then measure the maximum serous–mucosal distance, perpendicular to the lumen, without including abscesses or diverticula in measurement: In this case, measurement can be taken in colonic segments adjacent to them. Furthermore, if lumen is poorly visualized, thickness can be calculated measuring the serosa–serosa distance and then dividing the result in half (Fig. 1); (d) *grade of pericolic inflammation*: this finding manifests through the presence of hyperdense threads and hyperaemic vessels, which are increasingly less recognizable as the extent of inflammation increases. A system of grading allowed to qualitatively measure the level of pericolic inflammation (Fig. 2); (e) *presence and dimension of the peridiverticular or intramural abscess*: abscess was defined as a localized collection > 1 cm in diameter, of heterogeneous density (fluid/corpusculate), with or without air bubbles, delimited by a contrast-enhanced rim; maximum diameter was measured in horizontal plane.

### Statistical analysis

Categorical variables were summarized as counts and percentages and were compared using the Chi-square test (χ^2^) or Fisher’s exact test, in case of low frequencies. Continuous variables were summarized as means and standard deviations and were compared using the unpaired *t* test (Student *t* test), adjusted with Welch–Satterthwaite method or integrated with the Mann–Whitney U test, or ANOVA test in case of more than two groups. Univariate analysis using Cox regression models was employed to evaluate the effect of each predictor on diverticulitis recurrence over the 12-month follow-up period; multivariate analysis was used to evaluate correlation between CT predictors. The hazard ratio (HR) and 95% confidence intervals were calculated. Kaplan–Meier recurrence-free curves were created to graphically illustrate time-to-event rates throughout 1 year and compared using the log-rank test. The maximally selected rank statistics (MSRS) were used to identify the optimal wall thickening cutoff for the prediction of recurrence. In all analyses, the Pearson correlation coefficient was calculated. A *p* value < 0.05 was considered statistically significant.

Statistical analyses were performed with IBM SPSS Statistics software (version 29.0).

## Results

Demographic characteristics and diverticulitis-related CT findings of NR- and R-pts are summarized in Table 1. No statistically significant differences were found between the 2 groups regarding sex and age distribution. A trend was found in distribution of Hinchey Ib at the 1st episode, that was higher in R- than in NR-pts (30% vs. 12%), even if not statistically different. As far as parameters of diverticular disease severity were concerned, there were no differences between NR- and R-pts in overall number of diverticula and number of diverticula within 10 cm. In both groups, the most involved segment was rectosigmoid colon (78% and 81%), followed by the descending colon (47% and 31%). Ascending colon and caecum were much less involved, and no cases of transverse colon diverticulitis have been found in our study population. Mean length of the involved colonic segment (8.4 cm) was the same in two groups as well as the prevalence and mean diameter of the eventual abscess. Instead, a statistically significant difference was found regarding maximum wall thickness of the inflamed segment, R-pts showing a higher maximum parietal thickness than the other group (*p *< >0.001). A significant difference between the 2 groups was also found in peridiverticular inflammation with prevalent grade 4 in R-pts and prevalent grades 1 and 2 in NR group (*p *= >0.016).

**Table 1:** Demographic characteristics and diverticulitis-related CT findings of populations

	NR-pts (n = 33)	R-pts (n = 33)	*p-value*
Sex	M = 21 (64%)	M = 20 (61%)	0.800^†^
	F = 12 (36%)	F = 13 (39%)	
Age^§^	58 (12.2)	61 (14)	0.408*
Hinchey			0.071^†^
Ia	29 (88%)	23 (70%)	
Ib	4 (12%)	10 (30%)	
Multiple diverticula^§^	YES = 31 (94%) NO = 2 (6%)	YES = 32 (97%) NO = 1 (3%)	0.555^†^
	12 (7.2)	14 (7.3)	0.386^#^
Involved segment			0.595^†^
Rectosigmoid colon	26 (79%)	27 (82%)	
Descending colon	14 (42%)	10 (30%)	
Transverse colon	0 (0%)	0 (0%)	
Ascending colon	2 (6%)	1 (3%)	
Caecum	1 (3%)	1 (3%)	
Involved segment length (cm)^§^	8.4 (3.9)	8.4 (2.4)	0.669*
ABSCESS (mm)^§^	3 (6%)	9 (18%)	0.282^†^
	19	24.5	0.393^#^
Maximum wall thickness (mm)^§^	11.5 (2.6)	16 (4.2)	< 0.001^#^
Peridiverticular			0.016^†^
Inflammation			
Grade 1	10 (30%)	3 (9%)	
Grade 2	12 (37%)	7 (21%)	
Grade 3	7 (21%)	10 (30%)	
Grade 4	4 (12%)	13 (40%)	

# Student *t* test

* Mann–Whitney test

† Chi-square test (χ2)

§ Mean with standard deviation in parentheses

At univariate analysis with *Cox regression model*, significant predictors of diverticulitis recurrence were maximum thickness of colonic wall and grade 4 of peridiverticular inflammation: When thickness increases of 1 >mm, the hazard increases by 1.25 (1.15–136, *p *< >0.001) times; when peridiverticular inflammation is grade 4, there is a 344 times risk of recurrence than inflammation grade 1. Multivariate Cox regression showed that maximum thickness of colonic wall remained significant while grade 4 of peridiverticular inflammation reduced its predictive power (Table 2).

**Table 2:** CT predictors of recurrence in 1-year follow-up—univariate and multivariate cox regression analysis

	Univariate analysis		Multivariate analysis	
	Hazard ratio (95% CI)	*p*-value	Hazard ratio (95% CI)	*p*-value
Peridiverticular inflammation				
Grade 1	Reference	–	Reference	–
Grade 2	1.64 (0.42–6.34)	0.475	1.28 (0.33–4.99)	0.940
Grade 3	1.73 (0.94–12.41)	0.063	1.33 (0.33–5.41)	0.322
Grade 4	3.44 (1.51–18.71)	0.009	2.09 (0.55–8.01)	0.280
Wall thickness	1.25 (1.15–1.36)	< 0.001	1.23 (1.12–1.36)	< 0.001

**Fig. 3:**
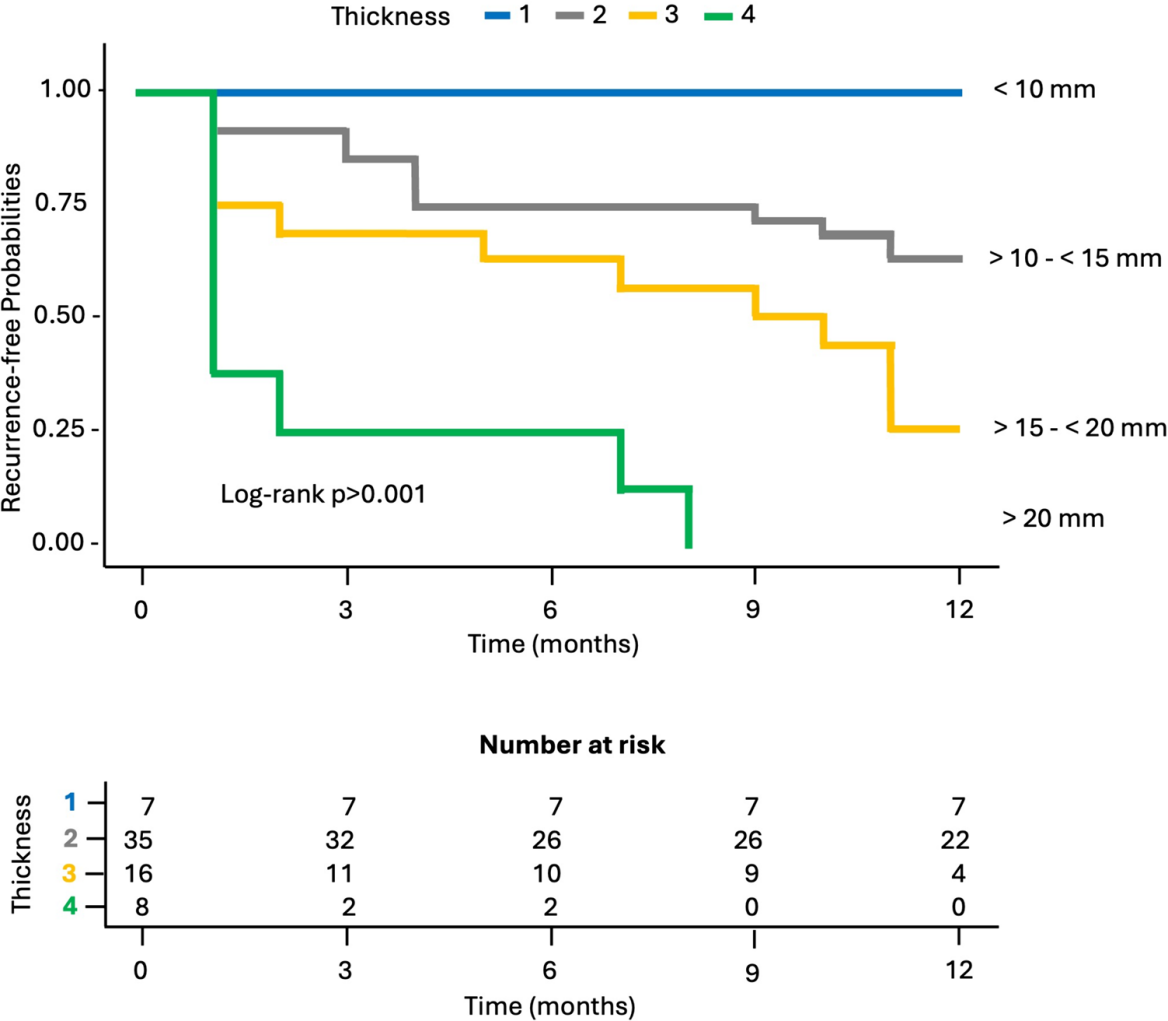
Kaplan–Meier plot for recurrence-free probability stratified by colonic wall maximum thickness groups during 1-year follow-up. Patients with a parietal thickness > 15 mm had a 12-month recurrence-free probability < 0,25 (log-rank test *p* < 0.001)

**Fig. 4:**
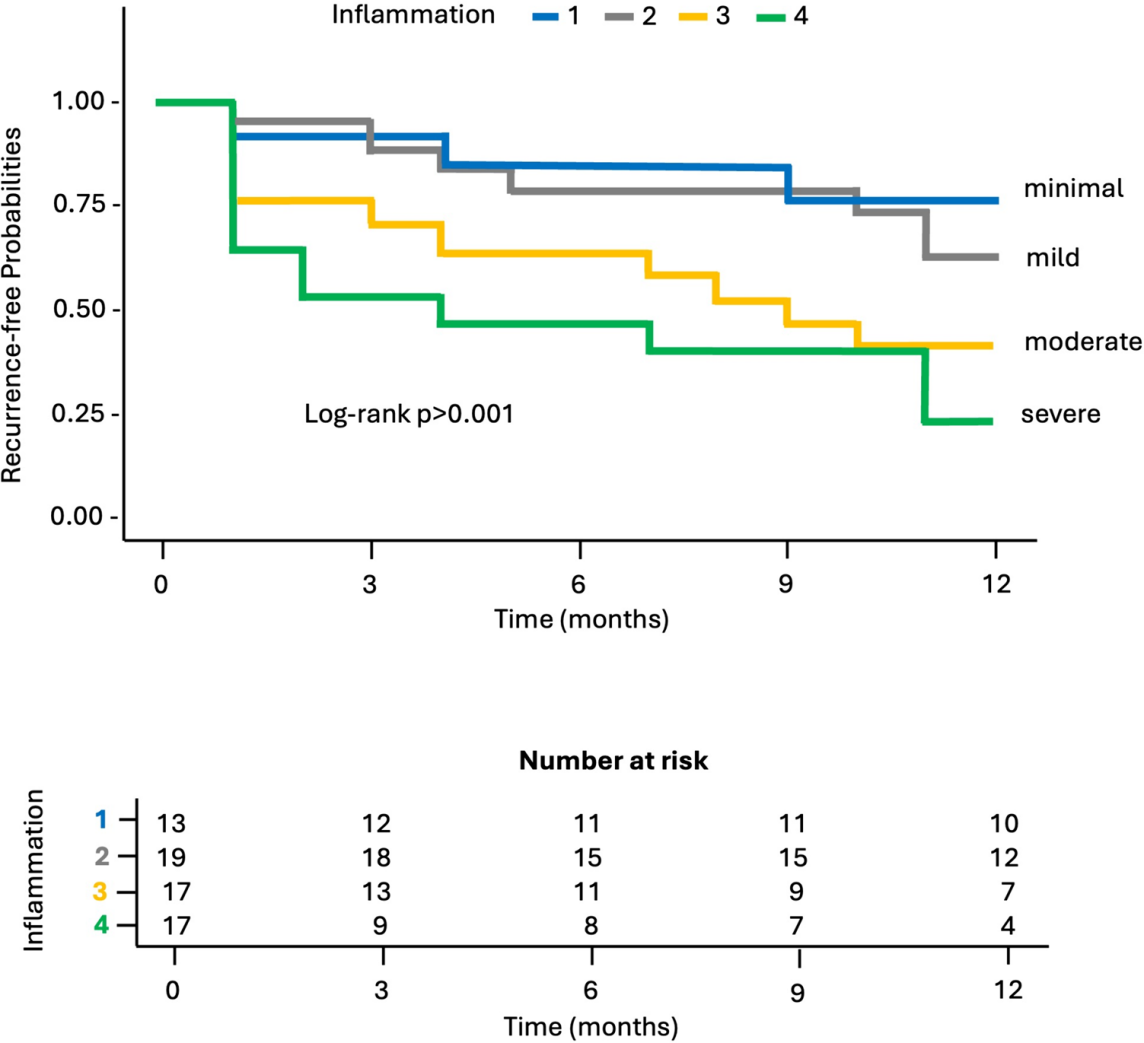
Kaplan–Meier plot for recurrence-free probability stratified by peridiverticular inflammation groups during 1-year follow-up. Patients with a grade 1 and 2 of peridiverticular inflammation had a significantly higher recurrence-free probability at 12 months than those with 3 and 4 grades (log-rank test *p* < 0.001)

*Kaplan–Meier plots* were produced for the significant predictors identified, to illustrate the relationship between the predictors and 1-year time-to-event rates. For wall thickness, continuous predictors were converted in categorical variables, and 4 groups were created: >10 mm, >10-<15 mm, >15-<20 mm, >20 mm. Figure 3 illustrates the recurrence-free probability stratified by colonic wall maximum thickness (Fig. 3). Similarly, for grade of peridiverticular inflammation, continuous predictors were converted in categorical variables, and 4 groups were created: minimal, mild, moderate, and severe (Fig. 4). Table 3 shows the estimated median recurrence-free months and 12-month recurrence-free rate for each significant predictor.

**Table 3:** Estimated median recurrence-free months and 12-month recurrence-free rate for each significant predictor

	Median recurrence-free months	12-Month recurrence-free rate
*Colonic wall thickness*		
< 10 mm	12 [11–12, 95% CI]	69% [48–99%, 95% CI]
> 10 mm, < 15 mm	9 [6–12, 95% CI]	42% [30–58%, 95% CI]
> 15 mm, < 20 mm	4,5 [4–9, 95% CI]	21% [11–40%, 95% CI]
> 20 mm	1 [2–8, 95% CI]	0%
*Peridiverticular inflammation grade*		
Grade 1	12 [4–12, 95% CI]	56% [36,5–87%, 95% CI]
Grade 2	11 [7–12, 95% CI]	39% [23,5–65%, 95% CI]
Grade 3	8,5 [4–12, 95% CI]	34% [22–53%, 95% CI]
Grade 4	4 [3–6, 95% CI]	15% [6,5–33%, 95% CI]

**Fig. 5:**
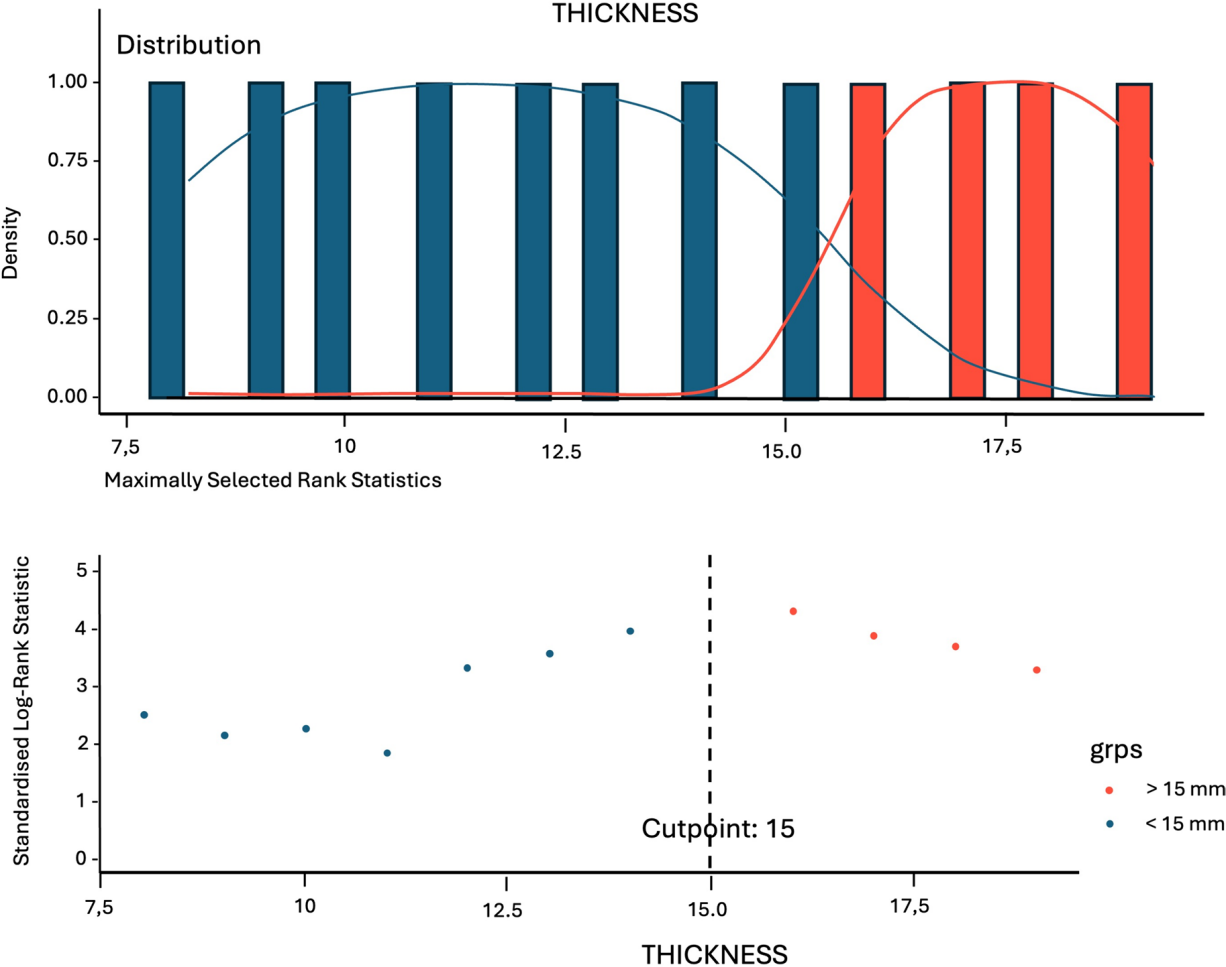
Scatter plot of maximally selected rank statistic shows the cutoff value of colonic wall thickness that predicts risk of recurrence

Using MSRS analysis, the optimal cutpoint for wall thickness for the prediction of recurrence was estimated and resulted to be >15 mm. Through curve fitting, which defines the “best fit” model of the relationship between thickness and recurrence-free rates, it was demonstrated that for thickness values <15 mm the HR remains almost constant, while for values >15 mm the curve slope begins to change and then tends to rise continuously upwards up to values of thickness =18 mm. This highlights that for high thickness values, patients present an increased risk to develop recurrence within 1-year follow-up period (Fig. 5).

**Fig. 6:**
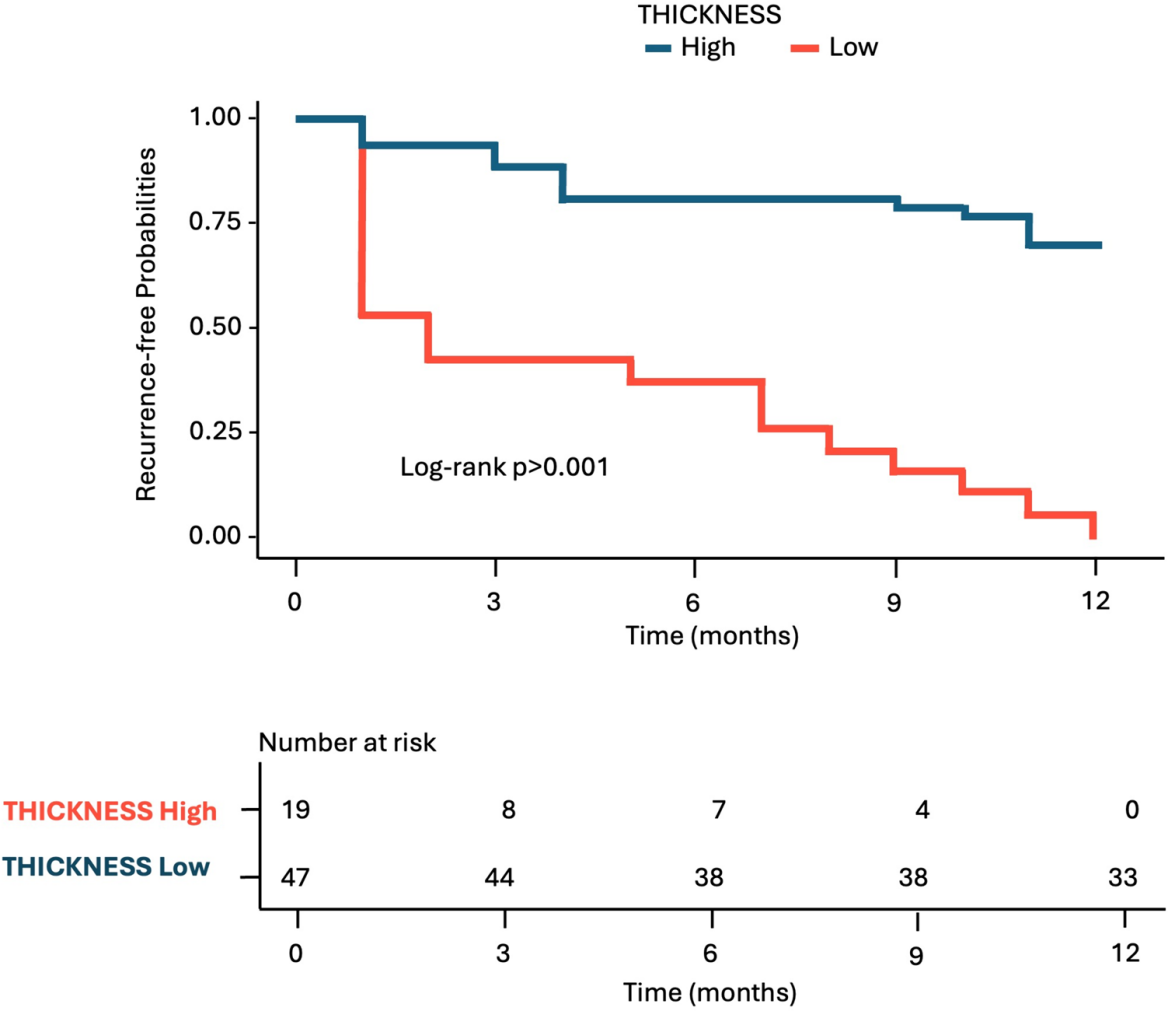
Kaplan–Meier curve according to the new cutoff value of colonic wall thickness (thickness high is > 15 mm and thickness low is < 15 mm)

Kaplan–Meier curve was reconstructed according to the new cutoff value to examine recurrence-free probability (Fig. 6). Recurrence-free rate of patients with thickness >15 mm was significantly higher than that of patients with thickness <15 mm (log-rank test, *p* < 0.001). In fact, the presence of thickness >15 mm causes a sixfold increased risk of recurrence compared to a thickness <15 mm (HR, 6.22; 95% CI, 3.05–12.67; *p* < 0.001).

Finally, in order to analyze the timing of the second event after the 1st episode of acute diverticulitis, diverticulitis-related CT findings were compared between recurrence within 90 days from first discharge (early recurrence, n = 13) after 90 days (late recurrence, n = 20). Results showed that mean parietal thickness was much higher in patients with early recurrence than those with late recurrence (18.5 mm vs. 14 mm; *p* < 0.001) and that grade 4 of peridiverticular inflammation was greater in first group (62% vs. 25% *p* = 0.036). Hinchey grade Ib at first event was more represented in the first group (46% vs. 20%, *p* = 0.011). Analyzing the relationship with Hinchey grade of the recurrence, patients with early recurrence showed a higher Hinchey on second CT (grade II: 31% vs. 5%; grade Ib: 62% vs. 45%; *p *= 0.002) and there was also a case of Hinchey III (Table 4). Even if no statistically different, there was a trend regarding mean length of inflamed segment, that was higher in patients with early recurrence (9 mm vs. 8 mm; *p *= 0.070).

**Table 4:** Comparison of CT findings in patients who developed acute diverticulitis < 90 days from first episode discharge vs. patients developing recurrence after 90 days

	< 90 Days (n = 13)		> 90 Days (n = 20)	*p*-value
Sex	M = 8 (62%)		M = 12 (60%)	0.988^†^
	F = 5 (38%)		F = 8 (40%)	
Age (years)^§^	60 (13.7)		62 (14.5)	0.694^#^
Maximum wall thickness (mm)^§^	18.5 (3.73)		15 (4.03)	< 0.001^#^
Peridiverticular inflammation				0.036^†^
Grade 1	1 (7%)		2 (10%)	
Grade 2	0 (0%)		7 (35%)	
Grade 3	4 (31%)		6 (30%)	
Grade 4	8 (62%)		5 (25%)	
Length (cm)^§^	9 (2.32)		8 (2.34)	0.070*
Hinchey 1° event				0.011^†^
Ia	7 (54%)		16 (80%)	
Ib	6 (46%)		4 (20%)	
Hinchey 2° event				0.002^†^
Ia	0 (0%)		10 (50%)	
Ib	8	(62%)	9	(45%)
II	4	(31%)	1	(5%)
III	1	(7%)	0	(0%)
ABSCESS (mm)^§^	38% (5)		5% (1)	0.025^†^
	22.4 (11.2)		35 (17)	0.335^#^

# Student *t* test

* Mann–Whitney test

† Chi-square test (χ2)

§ Mean with standard deviation in parentheses

## Discussion

The present study highlights that some CT parameters result in the statistical prediction of recurrence within 12 months from a first episode of uncomplicated acute diverticulitis and could be useful in identifying patients for whom guidelines recommend a conservative outpatient treatment that might require a more intensive management aimed to prevent recurrence of the disease. Patients with a recurrent attack of diverticulitis may be at risk for developing complications and needs for emergency surgery. In the past, it was reported that about one-third of all patients with acute diverticulitis will have a recurrent attack, often within 1 year [10]. However, a recent meta-analysis [9] found an estimated recurrence of 12.9% after an uncomplicated episode of diverticulitis.

In the present study, which strictly included only patients with a first episode of uncomplicated acute diverticulitis, two CT parameters have been identified as predictive factors on admission CT for recurrence of disease within 1 year, the maximum colonic wall thickness of the affected portion of the colon and the degree of peridiverticular inflammation. These parameters are coherent with those reported in a retrospective cohort study [17] that included acute diverticulitis independently from Hinchey classification score with a longer period of follow-up (5 years). The colonic wall thickness results to be the most predictive parameter that persist to be significant after multivariate analysis. To our knowledge, for the first time a cutoff was determined. The presence of thickness >15 mm causes a sixfold increased risk of recurrence within 1 year, with values of 18.5 mm being predictive of early recurrence within 90 days.

In turn, after multivariate analysis, peridiverticular inflammation has no longer a predictive power when associated with parietal thickness in accordance with a previous study [21] in which it was found that marked thickening of the mesenteric fat was not proportionated to the modest increase in the colonic parietal thickness. However, this finding differs from an other cohort study [17], in which predictive power of peridiverticular inflammation severity persisted in multivariate analysis. These discordances could be related to the different types of AD population studied as well as to the methods used to determine inflammation severity. In the present study, a visual grade of severity was used to make a qualitative evaluation while in other studies a mean region of interest (ROI) measurement was employed. Even with the use of ROI, the power of peridiverticular inflammation severity resulted discordant being predictive in some studies [17] but not in others [18]. In the latter study, the authors proposed measuring the largest area of inflammation using a ROI and the size of inflamed area in cm^2^.

Accurate measurement is difficult to obtain also when measuring length of involved colonic segment. None of the other CT parameters that have been reported to be predictive of recurrence was confirmed in the present study. Length of colonic inflamed segment was no related to recurrence similarly to Dickerson et al [17] that estimated the linear percentage of the inflamed segment. However, the length of involved segment might be predictive of early recurrence in uncomplicated AD with significancy barely missed in the present study probability to ascribe to the small number of patients included in the analysis. A previous study [22] evaluating CT features predictive of complications in AD divided patients into the same groups (<90 days and >90 days) found length of inflamed segment as a significant predictor of complications within 90 days. It has been demonstrated that the severity of the primary episode may be associated with similar severity at the following episode [23]. Further, in our study, also the presence of an abscess was not related to higher recurrence risk even if discordant with other studies [24]. Moreover, a study [23] correlates a primary episode with abscess (Hinchey Ib) with recurrence that shows abscess formation. In our experience, this outcome was not significant, but we found that early recurrence-pts developed on second CT a higher Hinchey grade than late recurrence-pts.

Finally, the previously reported risk factors for age [9] and male sex [17] were not confirmed in the present study.

Concerning the limitations of this study: (1) the population sample includes only patients recruited in the PDTA with a one-year follow-up, being the expression of an accurate selection and not representing the general population. However the extreme homogeneity of the group allows the data to be validated. (2) The results of this retrospective study required to be confirmed prospectively, an analysis already ongoing in the PDTA that continues to operate actively.

To our knowledge, to date, this is the first study that established a cutoff value for a predictor of recurrence of acute diverticulitis. Furthermore, in the literature there are not studies that have evaluated at the same time and in the same cohort CT predictors of recurrence and the influence of detected predictors on early and late recurrence. A precise estimate of the cutoff beyond which the risk of recurrences increases could be useful in clinical practice to establish which patients are eligible for a different therapeutic strategy and follow-up. Up to date, no treatments have proven efficiency in prevention of recurrence. Antibiotics treatment had no significant effect on the reduction of recurrence rates in AD [9, 15], and even elective surgery does not eliminate the risk of recurrence [24]. Some evidence that still need to be confirmed indicate that intermittent rifaximin associated with probiotics could prevent recurrence [23]. The possibility provided by the above CT predictive factors of recurrence of selecting a homogenous group of AD patients to tailor specific new therapeutic strategies represents an important clinical opportunity.

## Conclusions

The maximum wall colonic thickness and the grade of peridiverticular inflammation can be considered as predictive factors of recurrence. These factors, together with length of inflamed segment and Hinchey grade 1b at first episode of uncomplicated AD, may aid in selecting patients of our PDTA for a personalized therapy, potentially suitable for a treatment for prevention of recurrence, thus reducing health costs and inappropriate hospitalizations.

## Abbreviations

PDTA: The Italian acronym for integrated care pathway
ICP: Integrated care pathway
BMI: Body mass index
CI: Confidence intervals
CT: Computed tomography
HR: Hazard ratio
AD: Acute diverticulitis
ED: Emergency Department
NR-pts: Non-recurrence patients
R-pts: Recurrence patients
